# Cancer survivorship: understanding the patients’ journey and perspectives on post-treatment needs

**DOI:** 10.1186/s13102-024-00864-y

**Published:** 2024-04-12

**Authors:** Conor Hussey, Moira Hanbridge, Maura Dowling, Ananya Gupta

**Affiliations:** 1 School of Medicine, University of Galway, Galway, Ireland; 2 IPPOSI - Patient Education Programme in Health Innovation, Dublin, Ireland; 3 School of Nursing and Health Sciences, University of Galway, Galway, Ireland

**Keywords:** Cancer survivorship, Unmet needs, Symptoms, Personalised exercise and rehabilitation, Quality of life

## Abstract

**Background:**

Cancer treatments have many adverse effects on patient’s health leading to poor cardiorespiratory capacity, muscular- degeneration, fatigue, loss of strength and physical function, altered body-composition, compromised immune-function, peripheral neuropathy, and reduced quality of life (QOL). Exercise programs can significantly increase functional capacity when tailored to individual needs, thus improving health. Exercise interventions in cancer rehabilitation, when supported by appropriate nutrition can be effective in attaining a healthy weight and body-composition. The successful rehabilitation program should also include psycho-social education aimed to reduce anxiety and improve motivation.

**Methods:**

The current study aimed to collect information on the post-treatment needs of cancer patients including barriers and expectations facing them, their caregivers and their families through consultation in focus group interviews. Cancer survivors living in the Republic of Ireland were recruited from the University Hospital Galway, community-based cancer centres, cancer support groups and social media platforms to participate in the study and attend a focus group interview. The focus group discussions were designed to obtain information on the collective views of cancer survivors on relevant topics selected. The topics were developed in consultation with a patient and public involvement (PPI) group supporting the study. The topics list was circulated to all participants prior to the focus group. The interviews were audio recorded and transcribed verbatim. Focus group transcripts were analysed subjected to a thematic framework analysis using NVivo.

**Results:**

Thirty-six participants took part in 9 focus groups. Our analysis uncovered two main themes. The first theme ‘cast adrift with no direction’ was grouped into three sub-themes: *everything revolves around treatment*; *panic and fear*; and *what exercise should I be doing?* The second theme ‘everybody is different’ was clustered into two sub-themes: *side effects get in the way*; and *personalised exercise program.*

**Conclusion:**

The study highlighted the lack of information and support needed by patients living with and beyond cancer. The study also highlighted the need for a personalised exercise programme designed to target the individual patient symptoms that would be ideal for the mitigation of long term symptoms and in improving QOL.

**Supplementary Information:**

The online version contains supplementary material available at 10.1186/s13102-024-00864-y.

## Introduction

Cancer survivorship begins at the time of diagnosis and continues until end of life and is broadly referred to as *‘living with and beyond cancer’* [1]. According to the National Cancer Registry Ireland (NCRI), there are 22,640 new patients diagnosed with cancer every year in Ireland [1]. With the improvement in cancer treatment the rate of survival has improved significantly and is estimated to be 77–98% at 1 year and 63–92% at 5 years post treatment for most cancers. Cancers with greater than 80% survival rate at 5 years include cancers of the testis, prostrate, breast, melanoma, Thyroid and Hodgkin lymphoma. There were 173,000 cancer survivors living in Ireland in 2016 and it is predicted to exceed 200,000 in 2020 [1]. The National Cancer Strategy Ireland 2017–2026 [1] has outlined the need for the establishment of support services providing effective management of post-treatment health issues and improve quality of life (QOL) for cancer survivors [2, 3]. Cancer and its treatments such as surgery, radiotherapy, and chemotherapy have an adverse effect on patients’ health and results in reduced physical and functional capacity, psychological and social issues which negatively impacts their QOL [4]. Physical function or capacity is the individuals ability to perform activities of daily living which is significantly affected by cancer side effects. Psychological issues include fear and anxiety [4, 5]. Social issues can arise due to changes in relationship with friends and family resulting from the cancer diagnosis [4, 5]. In addition, patients recovering from cancer have an increased risk of developing a large number of chronic, co-morbid conditions and respond poorly to standard treatment making it difficult to treat such conditions [1, 4–8]. This leads to long-term burden of illness, long-term morbidity and increased risk of premature mortality in cancer survivors [5, 7–10].

National Cancer Strategy (NCS) 2017–2026 highlighted the gap in knowledge about the cancer survivorship needs of patients in Ireland and recommended the development of cancer survivorship services to be prioritised [1, 8]. The NCS proposed the ALLIES model for cancer survivorship for Ireland (2019) [11] which outlines the importance of a survivorship program that is integrated into the cancer care pathway [11–14]. The development of such a service should be based on research towards understanding patients’ perspectives in defining the post-treatment needs of cancer patients [1, 11]. The long-term illness suffered from cancer treatment varies in both symptoms and severity between patients [13, 14]. Hence, effective management not only requires long-term monitoring but also personalised or adaptive rehabilitation programming that is tailored to the specific needs of the patient.

The current study aimed to identify the post-treatment needs of cancer patients including information on the barriers and expectations facing them, their caregivers and their families through consultation in focus group interviews with current and past patients. The information obtained was analysed using a thematic framework. This qualitative analysis helped us to better understand the patients’ perspectives on post-treatment needs of cancer survivors and highlighted the key requirements for addressing the gaps in cancer survivorship in Ireland. Understanding patients’ opinions on post-treatment needs will help us to develop a personalised and adaptive exercise-based rehabilitation program that can be integrated into the cancer care pathway. Further, the information obtained can be used to build and provide resources in the form of written or online information and web-based support that can guide and empower patients to define their own post-treatment service choices and patients can then navigate their own survivorship pathway.

## Method

### Research study design

**Fig. 1:**
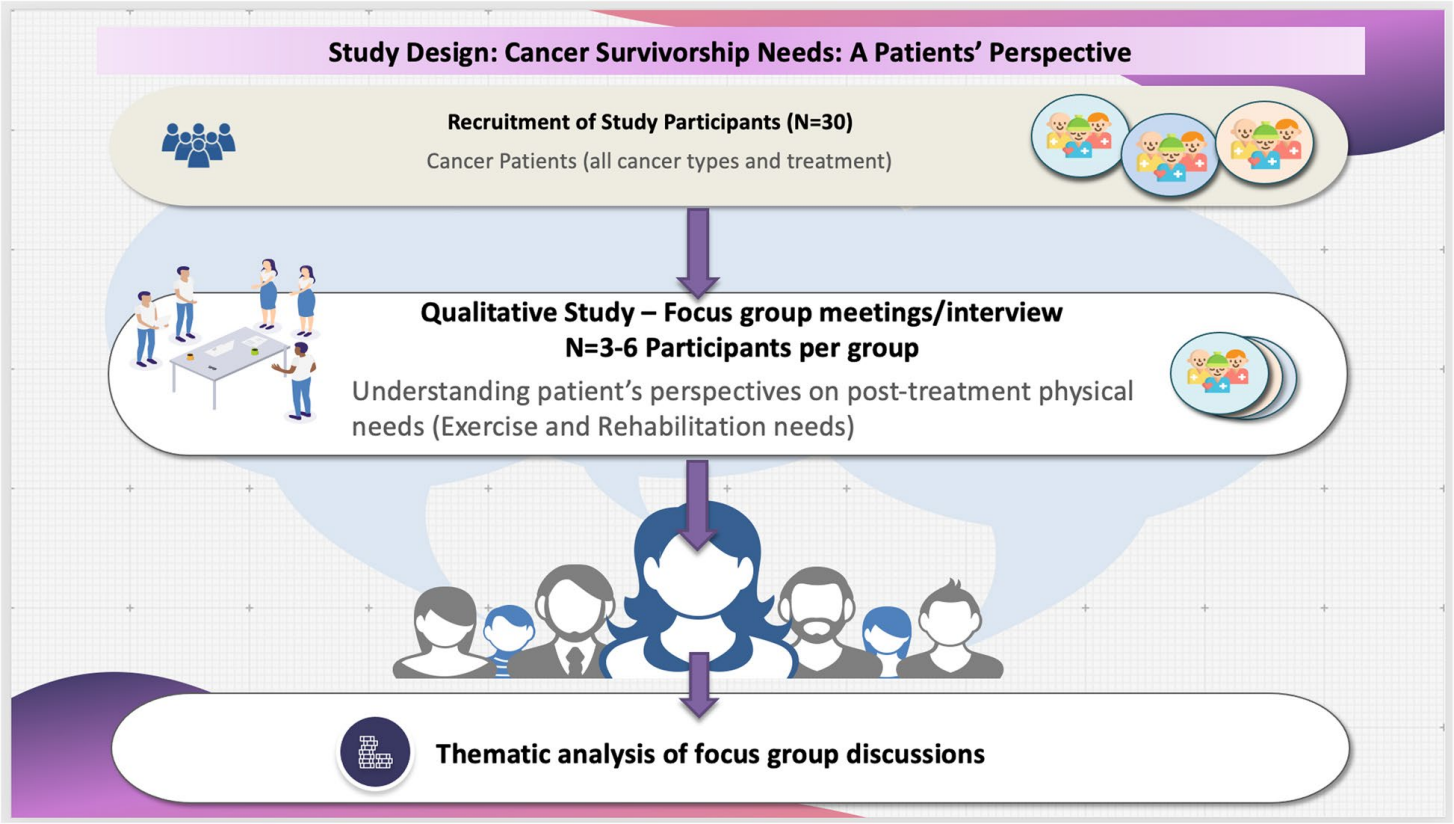
Research study design: Cancer Survivorship Needs – A Patients’ Perspective. The schematic diagram represents the design and work flow involved in the research study. Participants were recruited to the study from amongst patients treated at the UHG, through community-based cancer centres, patient advocate groups, PVCR (Patient Voice in Cancer Research) Dragon’s Den, IPPOSI (Irish Platform for Patients Organisations, Science & Industry) and social media. Patients were invited to participate in focus group meetings. A topics guide was provided to the patients in advance. The meeting was recorded and transcribed. The topics discussed were used to generate themes and sub themes in order to develop a framework for qualitative analysis

This qualitative study aimed to develop a comprehensive understanding of stakeholder perspectives on cancer survivorship needs through consultation with patients living with and beyond cancer. The main objective was to identify the post-treatment needs of cancer patients including information on long-term symptoms experienced, the barriers and expectations faced and information and resources needed to support their needs through rehabilitation. For this purpose a qualitative study was undertaken to elicit patients’ perspectives on post-treatment service needs of cancer survivors through focus-group interviews. The study involved recruitment of cancer survivors living in the Republic of Ireland were recruited from the University Hospital Galway (UHG), community-based cancer centres, cancer support groups and social media platforms to participate in the study and attend a focus group interview. A patient recruitment flyer was used to inform patients about the study. This was distributed to patients visiting the UHG oncology outpatient clinics, community-based cancer centres, and via the University of Galway website (https://www.universityofgalway.ie/can-react/) and social media posts. The focus group discussions were designed to obtain information on the collective views of cancer survivors on relevant topics selected. The focus group discussion guide consisting of topics to be discussed were developed in consultation with a patient and public involvement (PPI) group supporting the study. The PPI group was recruited from current and past patients treated at UHG and consisted of 5 members with lived experience of cancer and 2 cancer-care providers including an oncology nurse and a community-based cancer centre manager. The focus group topics guide (Appendix 1 – Supplementary information) was circulated to all participants prior to the focus group. The interviews were audio recorded and transcribed verbatim. Focus group transcripts were analysed subjected to a thematic framework analysis using NVivo. This study design is illustrated in the Fig. 1.

### Developing the focus group guide

**Fig. 2:**
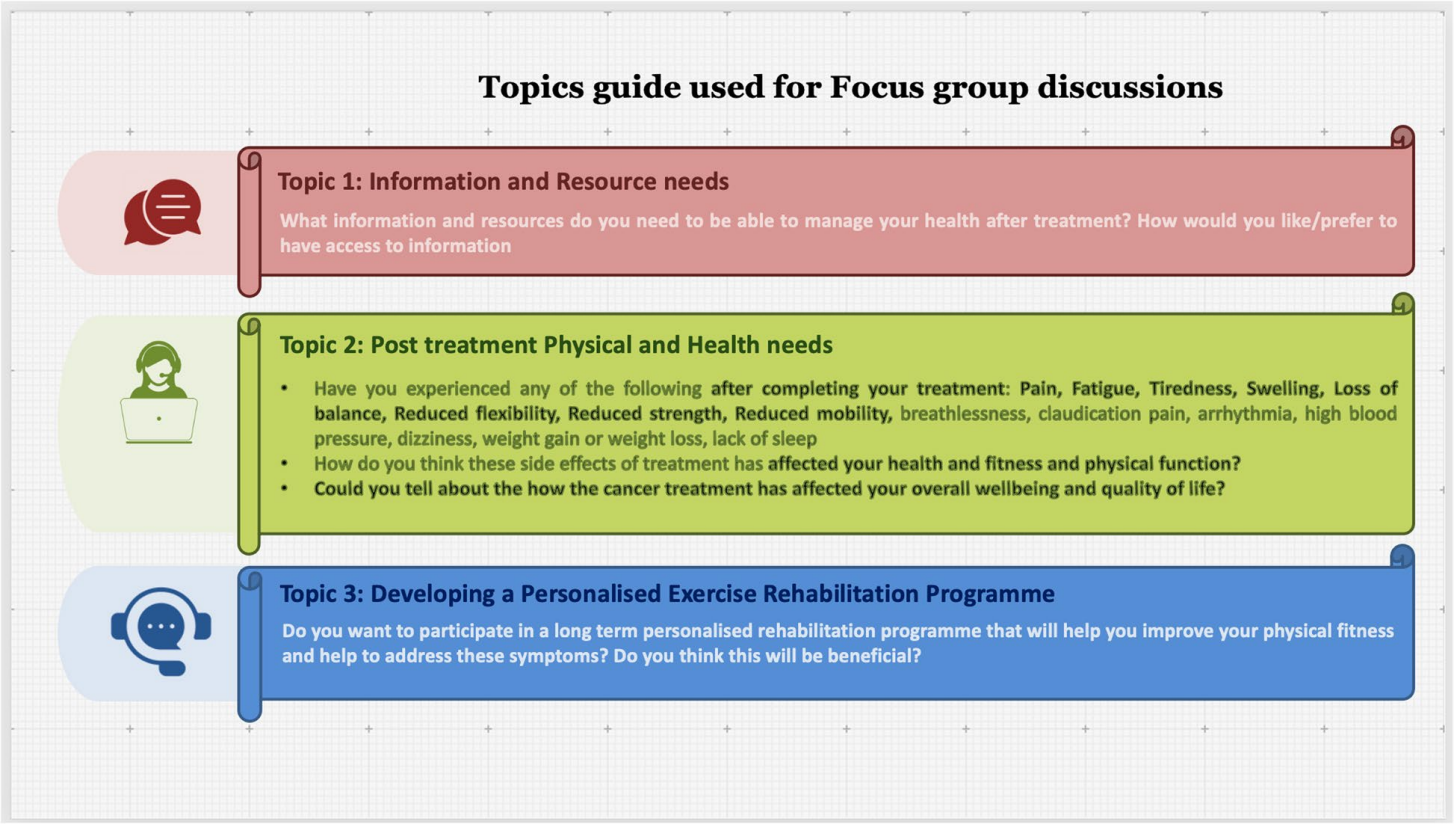
Focus Group Topics Guide. The schematic diagram outlines the topics guide used for the focus group interviews and summarises the prompts used to lead the discussions

The focus group discussions were designed to obtain information on the collective views of cancer survivors on relevant topics and the meanings that lie behind those views. The interview topic guide was generated based on previous research and tools available for assessment of cancer survivorship [15–18] in consultation with the PPI. A focus group guide consisting of information and outline of topics to be discussed (Fig. 2). (Also see Supplementary information- Appendix 1) was circulated to all the participants in advance. Informed consent for participation was obtained. The topics were useful in generating a rich overall understanding of participants’ experiences and beliefs.

Needs assessment measures are standardised tools that allow for the systematic identification of the areas for which patients perceive they require additional assistance. Such information allows for appropriate care to be developed and delivered to cancer patients in a timely manner. As the number of cancer survivors increases, it is imperative that our healthcare system addresses the unique and specific needs of this population. This requires cancer-survivor–specific needs assessment tools that are comprehensive, valid and reliable. Nine comprehensive needs assessment tools specific to cancer survivors were identified and reviewed. Most of the tools evaluated had undergone some form of psychometric assessment; however the extent and psychometric rigour of the measures was highly variable [15, 16, 18, 19]. Few of the measures identified had been evaluated for use in a clinical environment [16, 18, 19]. There is little empirical evidence to guide recommendations on the most appropriate process of conducting routine needs assessment with cancer survivors [15].

For the purpose of this study, we developed a modified topics guide based on the following published tools: Survivor Unmet Needs Survey (SUNS), short-form supportive care needs survey questionnaire (SCN_SF), Cancer Survivor’s Unmet Needs (CASUN) questionnaire [16–18, 20, 21]. The topics guide provided a framework for obtaining information on patient perceptions on the following key domains: (1) information and resource accessibility needs; (2) Comprehensive care needs: Physical needs, psychosocial needs, medical needs; (3) well-being, life change or QOL needs (4) financial needs (5) family/carer needs. For this study we focused on the first three domains only as these are the actionable needs that can be addressed through the resources to be developed as an outcome of this study. Additional questions were added to address key recommendations identified in the unmet needs assessment study in 2019 [8]. The topics guide included questions on individuals current physical fitness, participation in regular physical activity and how the side effects of treatment has affected their health and ability to participate in such activities. Physical activity encompasses all types of movement that requires a muscular action and expends energy. Exercise is an organised form of physical activity that is aimed to improve physiologic parameters such as cardiorespiratory capacity, muscle strength, balance and flexibility. These parameters contribute to improving the overall functional capacity of the individual and define an individual’s health related fitness. An exercise programme or intervention is the sum total of exercise planned for an individual over the span of 1 week which aims to improve one or more aspects of health related fitness. Of the 36 participants in the focus groups 28 patients were enrolled in a pilot 12 week personalised exercise programme. This programme included a baseline assessment of health related fitness, followed by an exercise prescription in which the Frequency. Intensity, Type and Time (FITT principles of exercise prescription) [22] of exercise performed is tailored to the individuals baseline fitness levels and aims to improve physiologic parameters that will help to enhance fitness. The exercise programme followed the ACSM guidelines for cancer survivors [23] and included a moderate intensity aerobic exercises, resistance and flexibility exercises. As part of the focus groups we also obtained feedback on the patients’ experiences following participation in this personalised adaptive exercise rehabilitation programme. Figure 2 illustrates the outline of the topics and prompts used to lead the focus group discussions. The detailed topics guide can be found in the supplementary information section (Appendix 1).

### Participants

Participants were cancer survivors living in the Republic of Ireland. A person who has received a diagnosis of cancer, until the end of their life, is defined as a cancer survivor according to the *National Cancer Institute* and *National Coalition for Cancer Survivorship* [1, 5].

Inclusion criteria were adult cancer patients in post-treatment recovery or living with long-term side effects/ illness resulting from cancer treatment, who were independently mobile, without communication and cognitive deficits, and discharged from hospital outpatient rehabilitation services, without other diagnosed neurological conditions. Participants were recruited from May–July 2021 and present and past patients in the Oncology department of UHG, associated primary-care centres, community-based cancer centres, cancer charities, through patient voice in cancer research (PVCR), IPPOSI and the community via social media posts. Table 1 in the results section summarises the patient characteristics.

**Table 1:** Participant Characteristics including patient demographics, disease type and treatment, Body composition and physical Activity levels are summarised

Participant Characteristics		
	Variable	Overall (*n* = 29)
Demographic profile	Age (year, mean (SD))	52.9 (7.2)
	Gender (female, No. (%))	28 (96.6)
	Time since diagnosis (years, mean (SD))	3.19 (2.6)
Cancer type (No. (%))	Breast cancer	22 (75.9)
	Multiple myeloma	2 (6.9)
	Prostate cancer	1 (3.5)
	Cervical cancer	1 (3.5)
	Brain cancer	1 (3.5)
	Kidney cancer	1 (3.5)
	Breast cancer, cervical cancer and Non-Hodgkin’s lymphoma	1 (3.5)
Treatment type (No. (%))	Surgery	26 (89.7)
	Radiotherapy	24 (82.7)
	Chemotherapy	23 (79.3)
	Hormonal therapy	15 (51.7)
	Targeted therapy	3 (10.3)
	Stem cell transplant	2 (6.9)
	Immunotherapy	1 (3.5)
Body Composition	BMI (Average, SD) Female	30.1 (5.9)
	BMI (Male participant)	28.1
Physical activity levels	Moderately Active (No. (%))	35%
	Inactive (No. (%))	64%
	(The Male participant was moderately active)	

### Data collection

Nine virtual focus groups with 36 participants were undertaken between June–September 2021. The demographics of the participants is summarised in Table 1. The number of participants in each focus group varied from 3 to 6. (Table s1 Supplementary Information). A minimum of 3 participants were planned for each focus group. The total number of focus groups was not determined at the outset and additional groups were organized until data saturation was achieved. Before their focus group, each participant was provided with a summary of the interview guide and the topics for discussion (Supplementary information – Appendix 1). The interview questions covered 4 main domains: 1. Information and Resource needs; 2. Physical health, and fitness needs; and 3. Comprehensive care and support needs 4. Personalised Exercise programme feedback. Interviews lasted between 55 and 125 minutes (average 75 minutes). The first focus group held on 1/07/2021 was attended by members of the PPI team which consisted of 5 members with lived experience of cancer and 2 cancer-care providers including one oncology nurse and one community-based cancer centre manager. Feedback was obtained after the meeting on the interview questions. This feedback was taken into consideration for all subsequent meetings.

The focus group discussions were led by MD who acted as a moderator and was attended by CH and AG. MD is a female researcher with expertise in cancer nursing and qualitative research. While MD was not involved in the exercise program and had not previously met any of the participants, during the focus groups it emerged that earlier in the year she had interviewed three participants by telephone for a different study. AG is a female exercise physiology researcher and was known to all participants. CH is a male physical exercise educator involved in devising personalised exercise programs and was known by many but not all of the focus group participants. The research team met following each focus group interview to discuss emerging themes, key issues arising, reflexive thoughts, and other observations. Notes from each meeting were kept for comparisons when discussing subsequent focus groups. All focus groups were video-recorded, anonymised and transcribed verbatim.

The COREQ (COnsolidated criteria for REporting Qualitative research) Checklist guided the study’s reporting [24]. Ethical approval for the study was granted by the Clinical Research Ethics Committee of University Hospital Galway (CREC Ref number: C.A. 2578). All study participants provided written consent before participation.

### Data analysis

All focus group transcripts were imported into QRS NVivo Version 12 [25] for the management of data analysis. Data analysis was undertaken independently by two authors (MD and CH), guided by the Framework Approach to analysis [26]. The Framework Approach combines both deductive and inductive approaches and was an appropriate choice because the data was based on focus group discussions framed by a semi-structured interview guide. Data analysis commenced with two authors (MD & CH) reviewing two focus group data for inductive themes and following discussion generating the a priori framework [26]. This framework guided data analysis across all nine focus groups and with further inductive themes added as needed. Regular meetings were held between MD and CH throughout the analysis to discuss reflexive thoughts and agree on the findings.

## Results

Nine virtual focus groups were undertaken between June and November 2021 with 36 participants in total (Table 1). The PPI team consisted of 7 participants and took part in the focus group discussions. The PPI team was composed on 5 breast cancer survivors, one oncology nurse and one cancer centre manager. One member of the PPI team attended each focus group meeting. The remaining 29 participants are described in Table 1. A participant was considered to be moderately active if they participate in a moderate intensity physical activity {i.e., requiring 3–6 metabolic equivalents (METs)}{Norton, 2010 #7398} for at least 30 minutes on 3 days in a week. All focus groups were moderated by MD assisted by two others (AG & CH). Each focus group was recorded and transcribed verbatim. The focus group (FG) meetings were organised between July–November in 2021and consisted of the following number of participants FG1 = 5, FG2 = 6, FG3 = 5, FG4 = 4, FG5 = 5, FG6 = 6, FG7 = 4, FG8 = 3, FG9 = 3. The codes linking each participant to the illustrative quotes used in the study is presented in Table s1.

The findings presented here represent the views and experiences of people living with different cancers, at different stages of their cancer trajectory, attempting to engage with physical activity. Our analysis uncovered two themes. The first theme ‘cast adrift with no direction’ (Table 3) was grouped into three sub-themes: *everything revolves around treatment*; *panic and fear*; and *what exercise should I be doing?* The second theme ‘everybody is different’ (Table 4) was clustered into two sub-themes: *side effects get in the way*; and *personalised exercise program.*

## Themes

The main findings of the study are organised into themes below.

### Theme: cast adrift with no direction

This theme reflects participants’ experiences on completion of active treatment. They felt shocked by the dramatic end of structured support when their treatment ended and left feeling lost. Ongoing feelings of fear were also an issue, especially fear of cancer recurrence. They had mixed experiences regarding health care professionals’ advice on exercise. When given information, it was mostly general, with little guidance on suitable exercise for their specific needs (Table 2).

**Table 2:** Theme: Cast adrift with no direction. Illustrative quotations

***Everything revolves around treatment***	*Everything revolves around your treatment. But also all of the people that have supported you like your friends, your family and everybody. They’re all there, all the time, while you’re going through your treatment. And the minute you’re finished your treatment it’s not that they completely disappear. But they, everybody wants to go back to being normal….So you’re kind of left you know there’s nothing.*[P2: FG3] […] *once you’ve finished your cancer journey you seem to be left out on a limb so I find yeah I really struggled* […] *I think yeah there’s definitely a lack of information you know everywhere once you finish your cancer journey.* [P7: FG4] *You’re dealt with by the professionals to get, you know to get rid of the cancer, give us the treatment, they’re excellent at what they do […] But it’s just you’re really dependent on them at that time […] when you leave them and you’re going back out into the world as such. That piece is missing*. [P4: FG4] “*I was chucked into a boat without any oars and shoved out into the ocean*” (P9; FG6). *You’re left kind of to your own devices, you feel quite isolated and alone.* [P6: FG4]
**Panic and fear**	*I’m just praying the days away to be honest, to get to next week. To feel a little bit better. You know you at a certain level of, before you start all of this but each step the way its getting worse, it’s going down and down, you’re losing strength, losing stamina, fitness, cardio, ability and you just at some point, you just feel so, I feel old, I feel weak* [P4: FG5] *I think the biggest fear, is the fear of recurrence and the fear of any little twinge, ache or pain is that something coming back ..*[P3: FG4] *I ended up in hospital a couple of weeks ago in agonising pain and straight away I had cancer back, it was in my bones, it was somewhere else, because your head goes there. […] Because straight away any kind of physical pain and you think it’s back, it’s here […] you become kind of hyper conscious and hyper responsive to every single feeling in your body. And I think that does, it’s very distracting, it’s all consuming at times* [P5: FG6] *I’m 6 years on now but I still obviously struggle with fear of reoccurrence* […] *I suffered really bad with depression when everything hit me afterwards […]* [P3: FG4] *I thought oh my god definitely going through the treatment would be the worst part but I think I’ve nearly found out that the post treatment [..] was just, for me I found it really difficult, you know what I mean, I was struggling*. [P3: FG5] *But like you know when you are struggling daily and whatever people just don’t get it […] you just get really withered with life […] when you are feeling so unwell and you can’t do things and you are pulled down, it’s very, very hard [..]* [P2: FG7]
***What exercise should I be doing***	*I certainly felt that there was just no support in any shape or form. And I’m not talking like emotional support or mental support. I’m just talking the physical, what should I be doing* […] *I wasn’t doing anything* [P13: FG8] […] *when I was having the radiation the radiation oncologist said if you could exercise that would help with the fatigue and I was thinking, he said I know you might think that’s impossible and I was thinking it is impossible. But there was no one out there to help or to know how to go about doing this exercise, how much or I felt I needed guidance […]* [P12: FG6] *I just feel in my experience there was such a lack of information around exercises, even the nurses had said to me exercise is very important for reducing recurrence etc, I was just still sent off on my own* [P3: FG4] […] *when I finished [chemotherapy] and I asked, I actually asked where do I go for support and help about exercise, diet to shift the weight I’d gained through chemo etc, etc,* etc. *And the nurse turned around, [..] She said we only do the chemo and we only deal with the chemo you know* […] [P9: FG6] *I suppose it was walking that I initially started with. But I suppose it maybe would’ve been helpful if there had been you know some kind of guidance as to what you could or couldn’t do. […]And there was very little of that.* [P1; FG3] *I asked my oncologist, you know finishing up [treatment] what’s the dos and don’ts and exercise was, she emphasised very strongly that it was something that you should do, yeah. But like that she quoted the WHO guidelines of, isn’t it 30, what is it 150 minutes a week* […] *but it literally was tiddle off now and do that yourself. I would have absolutely loved some kind of support like that, absolutely loved it.* [P10: FG1] […] *inside [hospital] they told me exercise, exercise, so my husband was alive at the time and I had a personal trainer and I was paying this fella 45 euro an hour I was going about three times a week. And sure I was going to him and I was going to the physiotherapist So then I tried the gym and then I injured myself in the gym. Then I said no, forget about it. So yeah I stopped to be honest with you because I was doing more harm than good.* [P1: FG7]

#### Everything revolves around treatment

In the sub-theme ‘*everything revolves around treatment’*, participants reflected on health care professionals’ primary focus on treatment and their sense of isolation on discharge, feeling ‘left out on a limb’ (P1: FG4). They used terms like ‘loss’ and felt they had fallen ‘between the cracks’ (P2: FG7) and struggled with the transition, not knowing what to do next to improve their sense of wellbeing: ‘being able to move and functionality is just, it’s missing’ (P1:FG6). This aspect of their journey permeated deeply into their overall experience*.*

Participants explained how upon completion of treatment, they yearned to return to how they felt pre-diagnosis. However, cancer and its treatment resulted in a myriad of physical and psychological side effects that made it extremely difficult to begin the process of restoring their health; ‘if you’re feeling a bit rubbish. It’s, you know it is much harder to (Laughs) talk yourself into going out and doing some exercise’ (P1:FG3). Participants wanted to kick-start their recovery by improving their lifestyle but there was no structured guidance; ‘there’s not a lot of structured follow up with regard to exercise and nutrition (P4:FG1). Some participants relied exclusively on cancer charities, such as the Irish Cancer Society for information. Others accessed online message boards; ‘searching the web myself and doing my own research’ (P2:FG8).

#### Panic and fear

A common experience shared across all focus groups was participants’ sense of panic and fear. Many descriptions suggest distress including ‘rolling panic attacks’ (P5; FG1) and ‘facing death’ (P3; FG1). Other frequent terms used included ‘depression’, ‘fear’, ‘isolated’, ‘feeling down’ and ‘paranoid’.

One reason explained for these feelings was related to a worry about deteriorating physical function’ ‘it’s a long time to lose strength, fitness and it’s very hard at an older age to actually regain that. That’s what kind of gets me. You know you at a certain level of, before you start all of this but each step the way it’s getting worse, it’s going down and down, you’re losing strength, losing stamina, fitness, cardio, ability and you just at some point, you just feel so, I feel old, I feel weak’ (P1:FG5). Participants were acutely aware of their decline in physical function but they also felt incapable of halting or reversing this decline due to a lack of support and guidance: ‘it’s all about prevent recurrence and you know keep yourself healthy but sure if nobody tells you the details it’s very frustrating’ (P2: PG4). Participants stated that a continued feeling of helplessness can lead to negative thoughts and depression in some instances. Moving on from treatment was for some, more difficult than the treatment itself; ‘to get through the treatment psychologically is as difficult as getting through it physically. And when you finish treatment picking yourself up and carrying on is far more difficult than the treatment itself’ (P4: FG3).

There was also a ‘fear of recurrence’ (P3; FG4), which resulted in a heightened awareness of their bodies ‘of any little twinge, ache or pain is that something coming back’ (P2; FG4). The cancer diagnosis caused participants to feel let down by their bodies. ‘It is frustrating when suddenly your body lets you down’ (P1:FG3). Regretfully, participants examined their past lifestyles to try and find the cause of their illness: ‘was I drinking too much, oh I smoked in my 20s, you know you do kind of look back and kind of start feeling guilty then about. And then start feeling guilty about things you’re doing now’ (P1:FG1). Unable to explain why they developed cancer, participants were intent on changing their lifestyle to reduce the likelihood of reoccurrence. However, without support or guidance, and ‘little emphasis’ put on activity levels (P5; FG4), participants felt stressed, anxious, and fearful in relation to their post-treatment lifestyle; ‘the mental side, so it was just a domino effect of kind of a negativity, just because of the lack of knowledge’ (P2:FG2).

The COVID-19 pandemic was also the source of fear for some participants. They explained their sense of isolation resulted in feelings of loneliness; ‘could it be the fact that we have covid at the moment and you can’t go out and see people?’ (P3; FG1). As vaccinations were rolled out and restrictions were lifted, participants were anxious about their immunity status: ‘I just don’t trust my immunity and there isn’t a lot of clarity either on people of what stage they’re at in their treatment, how many months after or how at risk they are or. So that definitely brings anxiety for me at the minute’ (P2:FG8).

#### What exercise should I be doing

Participants discussed their uncertainty on what exercise was suitable for them. The sub-theme ‘*what exercise should I be doing’* captures the lack of support, encouragement, and guidance participants experienced post-treatment during their quest to utilize exercise to maximize their recovery from cancer.

Participants were acutely aware of the positive impact exercise could play in their recovery from cancer; ‘exercise is so important’ (P2:FG1). They discussed how health care professionals promoted exercise as a tool to improve survivorship outcomes; ‘the nurses had said to me exercise is very important for reducing recurrence’ (P2; FG4) but expressed frustration with the vague nature of guidance given and inappropriate support or encouragement; ‘there was no one out there to help or to know how to go about doing this exercise, how much or I felt I needed guidance’ (P4; FG6); ‘would’ve been helpful if there had been you know some kind of guidance as to what you could or couldn’t do. Or what things were more beneficial than others’ (P4; FG3).

Feelings of isolation were expressed by participants with pre-diagnosis inactivity. They were being advised to change their lifestyle but left without appropriate support or encouragement: ‘I couldn’t do the kind of exercise I needed to do […] I had a fear of going for a walk with somebody. Because I couldn’t keep up and I was walking like somebody who was twenty years older than I am’ (P2.FG3). The enduring threat of cancer recurrence also added to this sense of isolation.

Fear of doing more damage than good when exercising was a major concern for them: ‘it’s the fear of doing yourself more damage…the fear, you’re told oh protect the arm, mind the arm’ (P5:FG6). A lack of guidance combined with treatment side effects, such as fatigue, forced them to choose to refrain from exercise: ‘the physical (side-effects) end of it for me was, made it quite difficult to exercise’ (P1:FG3).

In addition, lack of guidance resulted in feelings of regret and anger; ‘then I injured myself in the gym. Then I said no, forget about it. So yeah I stopped to be honest with you because I was doing more harm than good’ (P1: FG7). This was also an issue for those who had been regularly active prior to their cancer diagnosis. One participant was ‘very fit’ before diagnosis and had engaged in ‘high intensity training classes’ but now ‘couldn’t even attempt’ such level because of ‘head spinning’ and ‘dizziness’ (P6; FG4).

Even younger, fit participants were cautious and felt frustrated by a lack of support and guidance; ‘you have to be careful about what kind of exercises you’re going to do…even sort of trying to get clarity about that they [HC professionals] were a bit like, why are you asking that question. And I was like, well you know in terms of going back to doing exercise and you know if I’m going to go to the gym. Or even just you know I don’t know carrying heavy groceries or whatever. Like what’s okay and what can I do and what can I not do’ (P1; FG3).

### Theme: everybody is different

The second theme ‘everybody is different’ captures the ongoing side effects which impacted participants’ wellbeing and ability to exercise. They emphasized the unique needs of each person living with cancer, depending on their cancer and its treatment, and the importance of tailor-made instructions and support within a personalised exercise program (Table 3).

**Table 3:** Theme: Everybody is different. Illustrative quotations

**Fatigue and brain fog**	[…] *your wellbeing is impacted because you’re not in the mood and not in the form to be exercising, moving, you know you’re just exhausted you know and you’re tired and you’re fatigued and you’re wanting to feel the way you did pre all of this but you’re not feeling it.* [P6: FG4] […] *even from the beginning of diagnoses to going through treatment to getting into a post treatment stage, the fatigue is just horrendous […]Like even housework, I’m wrecked after a little bit of housework now these days, if I actually get to do it.* [P4: FG5] *I’m absolutely floored today I’m actually struggling really bad with fatigue today and just like heavy eyes and dull headache* [P14: FG7] […] *the fatigue is still there, the joint pain is still there, [...] I got into a place where it was like I know I need to do this* [exercise] *in order to feel better but in order to feel better I don’t feel I’m capable of doing this. And it was a vicious circl*e […]*.* [P13: FG8] *[…] cognitive, fatigue, I suppose it would go into fatigue but certainly my cognitive function, I think has been badly affected, certainly going back into a school setting, multi-tasking was extremely difficult. White noise, massive issue for me* [P4: FG2] *My ability to put thoughts together, my mind is really slow, extremely. And on the Tamoxifen was really, I couldn’t put a thought process together* [P2: FG5]
**Nerve pain and neuropathy**	*I remember the first time I experienced nerve pain, it scared me. Like it was very, very scary, it was very painful.* [P3: FG1] *So first of all I was in nerve pain, like severe nerve pain from when I came out of theatre and I ended up having nerve pain for 6 months afterwards. I had severe disability in my shoulder, like I couldn’t move, I was having nerve spasms, you know muscle tightness* [P5: FG1] *[…] there was all this weird nerve pain kind of going around into my shoulder and I got a bit of cording under my arm* [P1: FG1] *I suppose the biggest side effect that I’m struggling with and my treatment doesn’t end now till November but it’s the neuropathy. [..]I very much had it in my face and my hands and my feet. And just walking on carpet is akin to walking on broken glass. And that hasn’t quite changed even though an awful lot has returned to my fingers thank goodness*. [P2: FG6] *I have peripheral neuropathy, I mean I could keep on going with the side effects that I’ve had from it. My feet have completely changed position, they’ve changed in appearance. So it is extremely difficult to walk. And it’s all related back to the chemotherapy. Which is better the cure or the drug, you know, it’s hard to tell*. [P1: FG7]
**Menopausal symptoms**	*Not only hot flushes, vaginal dryness, mood changes, loss of libido, all those things which are not really, I think chemotherapy units are geared up to give you chemo and deal with neutropenia, nausea and vomiting. And I think there is definitely a deficit there dealing with menopausal symptoms in younger women. All women but particularly in younger women, it’s more problematic.* [P4: FG1] *I probably found the original, the kind of hot flushes, the hormonal treatments after chemo, that was probably one of the hardest things […] your sleep is off and you’re dealing with I suppose a lot of other stuff and suddenly you’re feeling like this kind of menopausal person, like 10 years ahead of what you ever anticipating to be like that.* [P2: FG1] *My biggest issue is the weight gain, I’ve gained like over 2 ½ stone. I know I was put into full blown menopause after my treatment but it totally was like unexpected* […] [P3: FG4] *I went into a very radical menopause when I started my chemotherapy. And I found that very isolating because no one of my age at the time was going through that and there was no support for that or there wasn’t even any information, nobody told me that this perhaps could happen to me.* [P4: FG4] *I had phenomenally aching joints, as in all over my body. And the physio said it is like arthritis. And I still have it, particularly in my feet and hands. We don’t know if its from the treatment or if it’s from, you know I’d be going through severe menopause now, we don’t know what it is. So again that was one I really struggled with […] I get a lot of hot sweats now and they really keep me awake at night actually […] yeah the nights, I wake up a lot at night with hot sweats and things like that.* [P1: FG9]

A variety of symptoms were experienced, some only experienced by a few participants and others experienced by many. The less common symptoms included cording (axillary web syndrome), pain, fibromyalgia, lymphedema, noise sensitivity, dental issues, systemic thrush, psoriasis flare up, nausea and vomiting, weight loss, and dry eyes.

#### Fatigue and brain fog

Cancer-related fatigue (CRF) was the most commonly mentioned physical side effect highlighted by participants. CRF diminished their ability to carry out everyday tasks, such as housekeeping; ‘I’m wrecked after a little bit of housework now these days, if I actually get to do it. [P1: FG5].

They wanted to exercise but were ‘pretty exhausted…My head wants to do everything but my body just won’t allow it’ (P2:FG8). CRF impacted their lives and their motivation to exercise; ‘you’re not in the mood and not in the form to be exercising’ (P4; FG4); ‘my body just feels like heavy and weak and like there isn’t a hope I’d be able to exercise today’ (P2; FG7). The constant nature of CRF prompted participants to ruminate and worry if they would ever return to their pre-illness energy levels. ‘I’ve had other bits and pieces but yeah that’s the one that’s kind of lingering’ (P6: FG6).

Participants commonly referenced their experience of CRF with cognitive fatigue. They used the terms ‘brain fog’ or ‘chemo brain’ and described how it negatively affected their ability to multitask or concentrate. Some also highlighted a decline in memory and reduced thought processes; ‘my cognitive function, I think has been badly affected, certainly going back into a school setting, multi-tasking was extremely difficult’ (P4; FG2). ‘I’ve gone completely more scatty than I was, I have to write everything down’ (P1: FG4). Participants referred to the psychological toll of constantly feeling fatigued. They were downhearted and despondent due to their inability to return to pre-illness energy levels: ‘[…] the fatigue and the brain fog and everything else, it’s just a nightmare (P5:FG6)’.

#### Nerve pain and neuropathy

Nerve pain as a consequence of breast cancer surgery and neuropathy from chemotherapy were experienced by some participants. Nerve pain was described as ‘very scary’ (P3; FG1) and a ‘severe disability (P5: FG1) affecting movement in shoulder and arm activities. At times, it was necessary to ‘tap’ the arm so the feeling would ‘come back’ (P5; FG3).

Numbness and pins and needles were experienced with feet ‘changed in appearance’ and extreme ‘difficulty’ in walking (P1; FG7). Fine motor movement was also affected; ‘I lost the pincher ability’ (P2; FG6) and holding items was a problem; ‘I can’t really hold anything here. I mean you know most of the stuff you need both of your arms and your hands, those are the things I can’t really do anymore’ [P5: FG3].

#### Menopausal symptoms

Menopausal symptoms were experienced related to chemotherapy and the anti-hormone treatment, tamoxifen. These symptoms were described as ‘more problematic’ in young women (P4; FG1) was ‘probably one of the hardest things’ (P2;FG1) to deal with post-treatment. Joint pain, ‘bone pain and aches’ (P4; FG4) affected participants’ ability to exercise and maintain a healthy weight; ‘I’ve gained like over 2 ½ stone…the joint pain was so bad it was inhibiting me from exercising’*.* (P3;FG4). Giving up tamoxifen was decided as the only option ‘for quality of life and being able to exercise’ (P3;FG4).

Weight gain was a common issue discussed and affected participants’ ability to exercise; ‘I’ve put on a lot of weight that’s causing the fitness issues I have at the moment’ (P2; FG3). Weight gain was hard to lose, even with engagement with exercise and a healthy approach to diet; ‘even with all the exercise, the walking, the rowing, I’m not able to get rid [of the weight]. I cook my food from scratch’ (P5;FG3). Hot flushes were also very troublesome with regular experiences of being ‘completely soaked’ (P2; FG7) and disturbed sleep at night which impacted on fatigue.

### Theme: personalised exercise program

This final theme reflected participants’ view on ‘everybody being different’ and the need for individualised exercise programs (Table 4).

**Table 4:** Feedback on the personalised exercise programme

***Personalised exercise program***	*[…] you can’t expect the same exercise program to be given just because we both have a cancer diagnoses in common. It’s not doing anybody any favours by both of us doing the same program. So I think that’s why it’s really important that its tailored and its progress orientated as opposed to goal orientated. And I think that is where that’s the key to it* [P2: FG1] *everybody is different, their levels of fitness are different. And as you say if they’ve had different surgeries and that, how debilitated they are after the surgery […] I think we have to give patients, you know instead of just saying exercise is important, we have to kind of get them assessed and see what exercise is appropriate for them*. [P4: FG1] […] *if you had to do it then, then with somebody watching you for a period of time. Just to get you going, do you know what I mean; I think that would be hugely beneficial.* (P2: FG3] *I think something like [..] a step by step program, you know with basic exercises I just feel would be really helpful. And actually breaking it down into like what you’re doing around the aerobic, the strength and flexibility*. [P2: FG4] *I find that this program is excellent and if this was available when you were coming out of your treatment it would be brilliant for people, just building confidence, targeting you and targeting what maybe different surgery because I know everyone has different cancers.* [P4: FG4] […] *if somebody was able to assess where I am at the moment with regards to the limitations and actually design something for me that’s not going to damage my or you know worsen any of my joints or mobility or something like that but actually improve it*. [P1: FG5] *I’m just really grateful that there’s something like this (Can react) that we can go forward with*. [P4: FG7] I’m enjoying the fact that you know have a piece of paper but you know I’ve my own notes written and so that I can actually do this. *** [Exercise physiologist] has made sure that I’m actually doing them right. So to me that’s a huge thing. I know what I’m doing and I’m hopefully, I can bring them on holidays with me. So that I won’t be behind the schedule you know. [P1: FG7] […] when I initially met *** [exercise physiologist] *he gave me the opportunity to go through my previous exercise history and gauge where I’m at with my fitness level. So it wasn’t just like a one for all program, which was brilliant. So yeah he took everything into consideration and came up with a fantastic plan*. [P2: FG8 ]

*** is used to mask the name of the exercise physiologist mentioned as part of the conversation

Participants were knowledgeable on the wider benefits of exercise. They highlighted its usefulness in alleviating anxiety and stress, reducing the risk of lymphedema, improving cognitive function, and improving symptoms of fatigue. They acknowledged the uniqueness of each cancer survivor with their varying levels of competencies in relation to exercise and activity and the side effects that impact exercise engagement; ‘everybody is different, their levels of fitness are different. And as you say if they’ve had different surgeries and that, how debilitated they are after the surgery […] I think we have to give patients, you know instead of just saying exercise is important, we have to kind of get them assessed and see what exercise is appropriate for them’ (P4: FG1).

For all these reasons, participants highlighted that cancer survivors need personalised exercise programs tailored to each individual’s ability and lifestyle. For those who had already started the personalised exercise program, feelings of success were shared. ‘I’ve my own notes written and so that I can actually do this’ (P1; FG7). These participants were motivated and felt accountable to achieve their best level of fitness in response to the different supports available to them on the program. ‘I can talk to somebody if the programme isn’t going well […] I’m doing something, because I’m, maybe its accountability, I don’t know. But it’s knowing somebody is there to talk to about the programme, if you need to talk about it’ (P2:FG3).

Participants utilized various forms of social media such as WhatsApp and Zoom to stay in touch with their exercise physiologist. This allowed them to exercise at home, at their convenience, whilst knowing support was always available. ‘So it wasn’t just like a one for all program, which was brilliant. So yeah he [exercise physiologist] took everything into consideration and came up with a fantastic plan (P2: FG8). These participants also stressed the need for any exercise program to progress gradually; ‘he [exercise physiologist] would be the one who would say to you start slow, we build up’ (P1; FG8). ‘I’ve really enjoyed the last few weeks. Because it kind of finally feels like I’m getting back a little bit of that fitness that I’ve lost. And I feel myself progressing’ (P1:FG3). This was a crucial aspect of the personalised program, as it allowed participants who may previously have been inactive, to gain confidence and incentive from achieving their achievable exercise target.

## PPI summary

The research study involved PPI input in the design of patient information flyers, study questions and topics to be discussed in the focus groups. A PPI group consisting of 7 members was set up consisting of patients living with and beyond cancer. PPI input was co-ordinated through meetings held over Zoom, with members of the research team and the PPI panel. PPI input included review, feedback and suggestions on the study flyers advertising the study, on the questionnaire and focus group topics guide designed for the focus groups and the lay summary describing the research.

Flyers/ patient information leaflet – input was given on design, content, structure and layout. Themes of lay readability and accessibility of these patient facing documents were our focus. The aim was to focus on the perspective of patient reading the flyer/patient information leaflet, to personalise it, as opposed to reflecting the research point of view. The PPI group helped to make all content relevant to the patient so that the participants can obtain a clear understanding of the aim of the study and contribute effectively towards the same.

Regarding the study title on the flyer, terms like “qualitative assessment” were advised against. It is not an accessible term to a lay audience. It was advised to “speak directly to the patient” as opposed to stating research intentions of the study, as is done in a research summary. The use of questions to attract readership was advised e.g. “are you a cancer patient or survivor? Would you like to improve your fitness? what are your post-treatment needs?” Questions suggested included- “what is involved for you?”, “what will we do with your input?” with the answers to be given in bullet point format. The tone was advised to be that of an invitation “you are invited to join our research study”. A simple format was advised- reducing word count, reducing level of detail given, use of bullet points and avoidance of use of all capitalisations. Suggestions were given on the imagery used. This feedback was taken on board by the research team and implemented, resulting in a much more patient friendly and lay accessible document.

Focus groups meetings- PPI input was given towards the planning of the focus groups, on what the post treatment needs were and what the relevant content would be for the questions used and the topics to be discussed in the focus group meetings.

The PPI panel took part in the first focus group meeting which served to iron out issues for the subsequent ones. PPI feedback was given on the structure and the chairing of the focus group. A suggestion was made that the questions could be supplied to the participants in advance, with an estimation of how much time would be spent on each one, to improve efficiency, flow and progress of the focus group meeting. It was suggested that it be made clear to the participants as to the type of information being sought.

It was clear that the PPI input was useful as it was taken on board and implemented. The experience of providing the input was fulfilling, as the research team were respectful of our position as patient advisors and were responsive to our feedback.

## Discussions

**Fig. 3:**
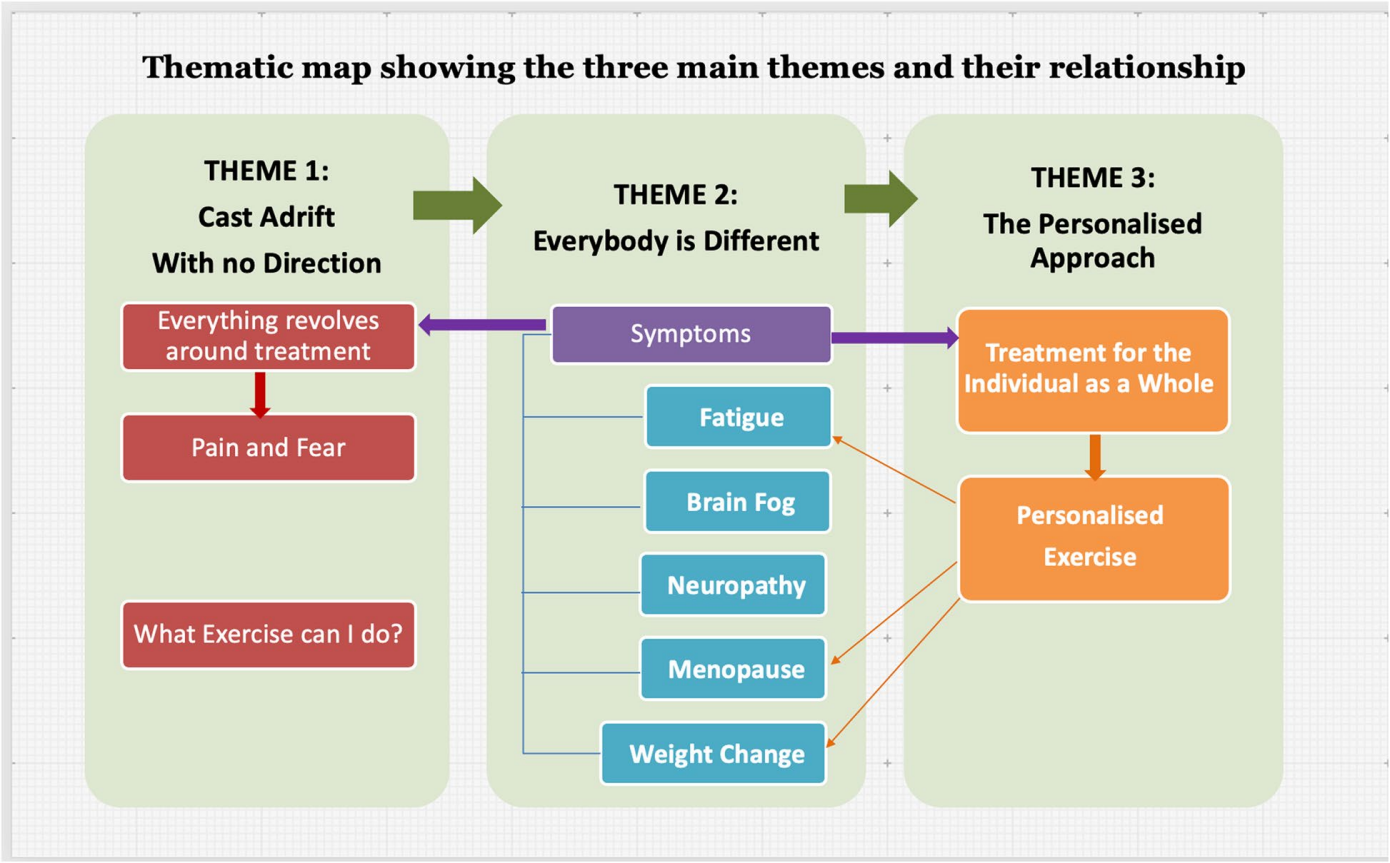
Thematic map showing the three main themes identified and their mutual relationship. The main themes were identified based on a thematic framework analysis using NVivo

The main findings of our study as illustrated by the thematic analysis shows that the patients have unmet post-treatment needs that can be broadly classified into (i) Information and support needs, (ii) Psycho-social needs, (iii) Physical needs and need for a “personalised” exercise programme (Fig. 3).

### Information and support needs

Our study findings highlight participants’ need for ongoing information in survivorship. Cancer survivors respect the authority and advice provided by their oncologists, who are ‘critical to the effective promotion and durable uptake of physical activity among survivors of cancer’ ([27]. However, doctors may not always be aware of the PA guidelines [28] to be able to provide appropriate guidance to patients. Moreover, PA guidelines differ between countries [29, 30]. Also constraints of time and resources make it difficult for clinicians to provide the required information and support to patients.

Participants in our study also revealed a lack of structured and accessible information on nutrition and exercise for cancer patients undergoing treatment. Cancer patients’ frustration over the vague information on exercise and physical activity provided by health care professionals’ has also been reported in a recent Italian study [31]. It is known that oncology health care professionals are aware of the importance of physical activity in cancer care, and many report promoting physical activity to their patients [32, 33]. However, many health care professionals also admit to not discussing the importance of exercise with cancer patients in their care [34]. The reasons for this are complex and related to time constraints during consultations, safety concerns, lack of knowledge about physical activity and not knowing or having access to a proper pathway for referral to qualified exercise professionals [31–35]. Moreover, in the context of treatment settings where a biomedical disease-focused model of care dominates, the emphasis is on supporting patient through managing their treatment, and opportunities to provide physical activity advice may be limited [36].

### Psycho-social needs

Participants stated that following diagnosis and treatment there was a persistent feeling of helplessness which can lead to negative thoughts leading to fear, anxiety and depression. This feeling persisted even after completion of treatment. Moving on from treatment for some patients was more difficult than the treatment itself*; ‘to get through the treatment psychologically is as difficult as getting through it physically’*. Such a consistent feeling of fear and anxiety was suffered by most participants long beyond the completion of treatment which in turn highlighted the need of continued psycho-social support during survivorship. These findings were similar to what has been previously recognised [5, 37].

### Physical needs and the need for developing a “personalised” exercise intervention

Physical interventions can benefit cancer patients by improving their functional capacity by reducing cancer-related pain and fatigue [38–40] Psychosocial interventions, including psycho-education and cognitive behavioural therapy (CBT), aim to improve psychological functioning and reduce symptoms of fear, depression, and anxiety [41, 42]. Most rehabilitation programmes attempt to target either physical *or* emotional symptoms. There are very few multidimensional rehabilitation programmes (MDRPs) that attempt to target both physical and psychological symptoms in cancer patients [3]. It has been proposed that MDRPs can provide people with information enabling the self-management of their care [43]. Consequently, such MDRPs have been implemented for patients suffering from chronic ailments [44–46]. However, due to a lack of sufficient research evidence, a small number of feasibility studies, variability between study protocols as well as the type and duration of interventions, and outcome measures used, the benefits and efficacy of MDRPs in cancer survivors are unknown [3, 47].

The long-term illness caused by cancer treatment varies in both symptoms and severity between patients. Hence, effective management requires long-term monitoring and a personalised/adaptive rehabilitation programme tailored to the specific needs of the patient. An adaptive intervention is delivered through stages, allowing monitoring and readjusting of treatment modalities according to individual progress. This adaptability requires a planned sequence of stages in the delivery of the intervention; each stage can be adapted to individual responses, and hence multiple personalised decisions can be made throughout the course of the intervention. The findings of a recent Cochrane Review [3] highlight a lack of coherent information from multidisciplinary adaptive rehabilitation programmes, due to wide variations in methods/study protocols, the nature of the interventions, and the outcome measures used. Therefore, a novel approach is required. Adaptive interventions are key to personalised medicine and are of particular significance in rehabilitation, where adaptability leads to optimised outcomes.

Our study has revealed participants’ ongoing symptom burden in survivorship affecting their quality of life and ability to engage in physical activity. Patient characteristics affect health care professional’s decision to recommend physical activity [48]. While health care professionals report willingness to recommend participation in physical activity when patients have side effects or appear to have a low affinity for such activity (i.e. are overweight or unfit), they are simultaneously hesitant to recommend physical activity to patients whose general health is poor [32, 48].

The study further highlighted the need for survivorship support to be integrated as part of the comprehensive care needs for a patient. Patients expressed the urgent need for receiving information and education regarding the benefits of exercise interventions in the management of treatment symptoms upon diagnosis. Participants also highlighted the need for building support services that can provide long term exercise and nutritional guidance. Participants concluded that the ideal location of these services would be in a community setting. Uniformity in delivery and accessibility of these services was also highlighted to play an important role.

A need for developing interventions that were tailored to the individuals and took into account all the symptoms and side-effects that they were experiencing would be most beneficial. The participants in our study shared the positive effects of the personalised exercise programme on relieving symptoms and reducing fatigue. The exercise was tailored to the individuals current level of fitness, symptoms and side effects. This enabled all participants to take part and. Participants received regular updates on their progress and felt that their symptoms became manageable. Participants felt that they were making progress. This feeling of confidence helped participants to comply with the programme. These were crucial aspects of the personalised program, as it allowed participants who may previously have been inactive, to gain confidence and incentive from achieving their personalised exercise targets and see reduction in their symptoms and side effects. Cancer patients’ perceived benefits of exercise is widely reported [31], and physical therapists are ideally placed as valuable members of the multidisciplinary team delivering individualised exercise programmes [49, 50]. Going forward, it will be beneficial if all multidisciplinary teams involved in providing long term care or cancer survivors should include a physical therapist or a clinical exercise physiologist. There is also a need for design and delivery of such a long term support programme through the community based cancer care centres.

**Fig. 4:**
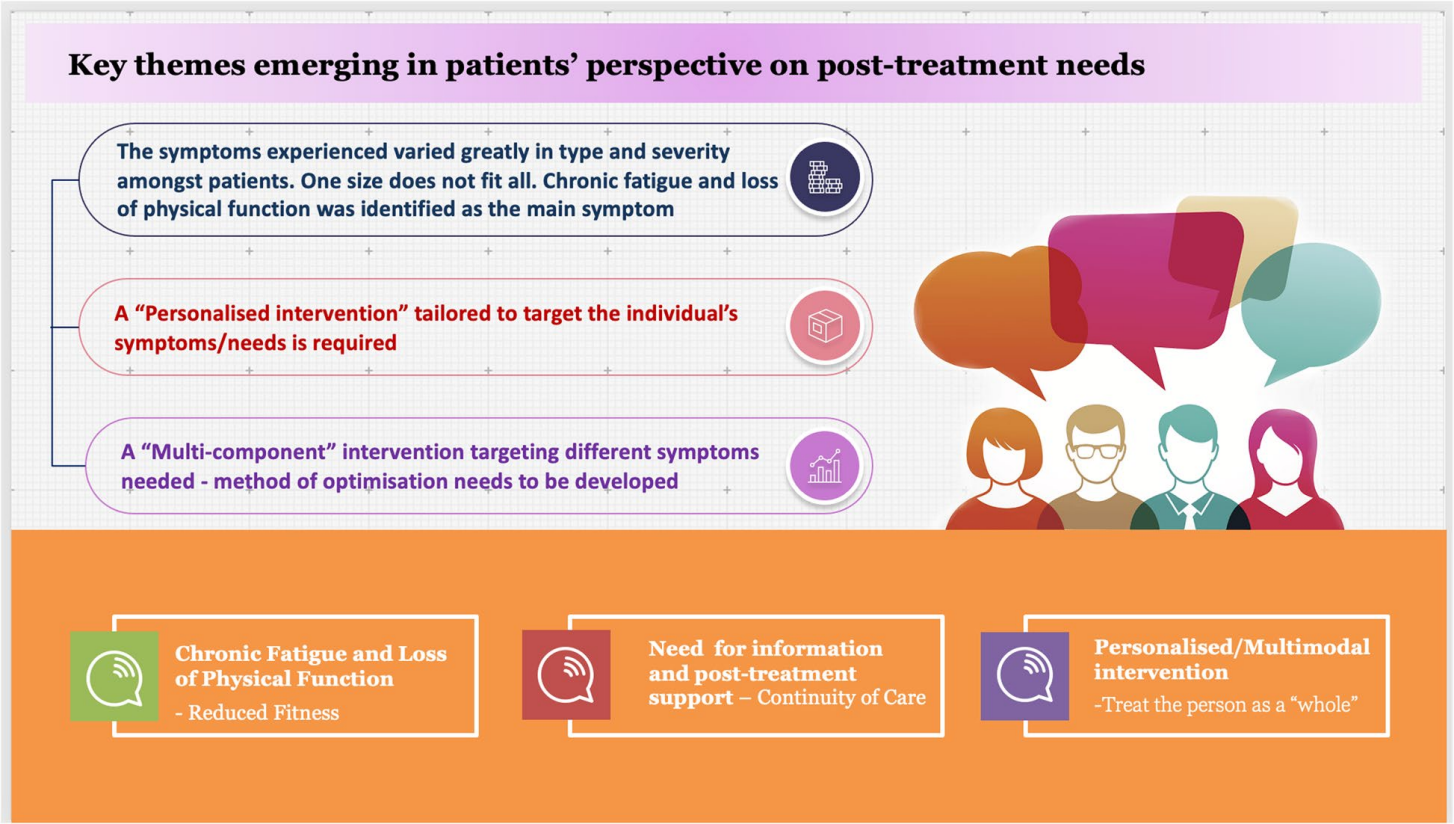
Key themes emerging in patients perspective on post-treatment needs

The patients perspectives of the key themes emerging are summarised in Fig. 4.

## Conclusions

The study had the overall objective of underpinning the post treatment and long term needs of cancer survivors. The main findings of our study as illustrated by the thematic analysis shows that cancer patients have unmet post-treatment needs that can be broadly classified into (i) Information and support needs, (ii) Psycho-social needs, (iii) Physical needs and need for a “personalised” exercise programme that can reduce the impact of the immediate and long term side effects. The strength of the study was in the clear methodology used and PPI involvement in study design and in analysing and interpreting the results. The emerging themes clearly identified the lack of information and support needed by patients in living with and beyond cancer. Although the study had a small cohort size and consisted of primarily female patients the key concerns around lack of information and support seemed to affect most patients irrespective of disease severity and treatment. Also pain and fatigue appeared to be the most debilitating side effects. Patients also highlighted the need for exercise guidance. The study highlighted the patient’s opinion on the effectiveness of a personalised exercise programme in the mitigation of long term symptoms and in improving QOL. There is an urgent need for design and delivery of such a programme and patients felt that this could be best delivered through the community based cancer centres.

### Supplementary Information

**Supplementary Material 1.**

**Supplementary Material 2.**

**Supplementary Material 3.**

## Data Availability

No quantitative datasets were generated or analysed during the current study. The qualitative datasets generated during the focus group interviews and analysed during the current study are not publicly available due to GDPR regulations but are available from the corresponding author on reasonable request.

## References

[1] Mullen L, Hanan T (2019). National Cancer survivorship needs assessment: living with and beyond cancer in Ireland.

[2] Mishra SI (2014). Are exercise programs effective for improving health-related quality of life among cancer survivors? A systematic review and meta-analysis. Oncol Nurs Forum.

[3] Scott DA (2013). Multidimensional rehabilitation programmes for adult cancer survivors. Cochrane Database Syst Rev.

[4] Mayer DK, Nasso SF, Earp JA (2017). Defining cancer survivors, their needs, and perspectives on survivorship health care in the USA. Lancet Oncol.

[5] Ivers M, Dooley B, Bates U (2009). Focusing on survivorship- improving our knowledge of life after Cancer.

[6] Egan MY (2013). Rehabilitation following cancer treatment. Disabil Rehabil.

[7] Ivers M, Dooley BA, Bates U (2009). Development, implementation and evaluation of a multidisciplinary cancer rehabilitation programme: the CANSURVIVOR project: meeting post-treatment cancer survivors’ needs.

[8] O’Connor M, O’Donovan B, Drummond F, Donnelly C (2019). The unmet needs of cancer survivors in Ireland: a scoping review.

[9] Gegechkori N, Haines L, Lin JJ (2017). Long-term and latent side effects of specific Cancer types. Med Clin North Am.

[10] Fow NR (1996). Cancer rehabilitation: an investment in survivorship. Rehab Manag.

[11] Hegarty JMA, Hanan T, O’ Mahony M, Landers M, McCarthy B, Lehane E, Noonan B, Fitzgerald S, Reidy M, Saab MM, Corrigan M, Mullen L (2018). Acute sector Cancer survivorship Services in the Irish Context.

[12] Moore AR (2015). Public health action model for Cancer survivorship. Am J Prev Med.

[13] Kline RM (2018). Long-term survivorship care after Cancer treatment - summary of a 2017 National Cancer Policy Forum Workshop. J Natl Cancer Inst.

[14] Stout NL (2019). Long-term survivorship care after Cancer treatment: a new emphasis on the role of rehabilitation services. Phys Ther.

[15] Pearce NJ, Sanson-Fisher R, Campbell HS (2008). Measuring quality of life in cancer survivors: a methodological review of existing scales. Psychooncology.

[16] Campbell HS (2014). Development and validation of the short-form survivor unmet needs survey (SF-SUNS). Support Care Cancer.

[17] Campbell SH (2014). Measuring the unmet supportive care needs of cancer support persons: the development of the support person's unmet needs survey--short form. Eur J Cancer Care (Engl).

[18] Campbell HS (2010). Psychometric properties of cancer survivors' unmet needs survey. Support Care Cancer.

[19] Campbell HS (2009). The cancer support person's unmet needs survey: psychometric properties. Cancer.

[20] Hall A (2014). The survivor unmet needs survey (SUNS) for haematological cancer survivors: a cross-sectional study assessing the relevance and psychometric properties. BMC Health Serv Res.

[21] Hodgkinson K (2007). The development and evaluation of a measure to assess cancer survivors' unmet supportive care needs: the CaSUN (Cancer Survivors' unmet needs measure). Psychooncology.

[22] Bushman BA (2018). Developing the P (for Progression) in a FITT-VP Exercise Prescription. ACSM's Health Fit J.

[23] ACSM. https://www.acsm.org/docs/default-source/files-for-resource-library/cancer-infographic-sept-2022.pdf*. *Accessed June 2021.

[24] Tong A, Sainsbury P, Craig J (2007). Consolidated criteria for reporting qualitative research (COREQ): a 32-item checklist for interviews and focus groups. Int J Qual Health Care.

[25] NVivo, NVivo (released in March 2020), QSR International Pty Ltd. 2020. https://www.qsrinternational.com/nvivo-qualitative-data-analysis-software/home. Accessed Sep 2021 - Sep 2023.

[26] Gale NK (2013). Using the framework method for the analysis of qualitative data in multi-disciplinary health research. BMC Med Res Methodol.

[27] Hardcastle SJ, Cohen PA (2017). Effective physical activity promotion to survivors of Cancer is likely to be home based and to require oncologist participation. J Clin Oncol.

[28] McCourt O (2021). Physical activity during and after Haematological Cancer treatment: a cross-sectional survey of Haematology healthcare professionals in the United Kingdom. J Multidiscip Healthc.

[29] Spence JC (2021). Determinants of physical activity among adults in the United Kingdom during the COVID-19 pandemic: the DUK-COVID study. Br J Health Psychol.

[30] Spence RR (2020). Physical activity and exercise guidelines for people with Cancer: why are they needed, who should use them, and when?. Semin Oncol Nurs.

[31] Avancini A, et al. Exercise levels and preferences in Cancer patients: a cross-sectional study. Int J Environ Res Public Health. 2020;17(15) 10.3390/ijerph17155351 PMC7432474 32722265

[32] Haussmann A (2018). What hinders healthcare professionals in promoting physical activity towards cancer patients? The influencing role of healthcare professionals' concerns, perceived patient characteristics and perceived structural factors. Eur J Cancer Care (Engl).

[33] Keogh JW (2017). Benefits and barriers of Cancer practitioners discussing physical activity with their Cancer patients. J Cancer Educ.

[34] Alderman G (2020). Health care Professionals' knowledge and attitudes toward physical activity in Cancer patients: a systematic review. Semin Oncol Nurs.

[35] Cantwell M (2018). Healthcare professionals' knowledge and practice of physical activity promotion in cancer care: challenges and solutions. Eur J Cancer Care (Engl).

[36] Karvinen KH (2017). Evaluation of online learning modules for improving physical activity counseling skills, practices, and knowledge of oncology nurses. Oncol Nurs Forum.

[37] NE A. Cancer Care for the Whole Patient: Meeting Psychosocial Health Needs. Washington (DC): National Academies Press (US); 2008. The Psychosocial Needs of Cancer Patients. Institute of Medicine (US) Committee on Psychosocial Services to Cancer Patients/Families in a Community Setting; Adler NE, Page AEK, editors. Available from: https://www.ncbi.nlm.nih.gov/books/NBK4011/, 2008. 1. Accessed June 2021. 20669419

[38] Kessels E, Husson O, van der Feltz-Cornelis CM (2018). The effect of exercise on cancer-related fatigue in cancer survivors: a systematic review and meta-analysis. Neuropsychiatr Dis Treat.

[39] Matsugaki R (2018). Immediate effects of exercise intervention on cancer-related fatigue. J Phys Ther Sci.

[40] Hussey C, Gupta A (2022). Exercise interventions to combat cancer-related fatigue in cancer patients undergoing treatment: a review. Cancer Investig.

[41] Daniels J, Sheils E (2017). A complex interplay: cognitive Behavioural therapy for severe health anxiety in Addison's disease to reduce emergency department admissions. Behav Cogn Psychother.

[42] Kucherer S, Ferguson RJ (2017). Cognitive behavioral therapy for cancer-related cognitive dysfunction. Curr Opin Support Palliat Care.

[43] Corner E (2015). Rehabilitation in critical care: barrier, hurdle or brick wall?. J Intensive Care Soc.

[44] Jolliffe JA (2001). Exercise-based rehabilitation for coronary heart disease. Cochrane Database Syst Rev.

[45] Khan F (2008). Multidisciplinary rehabilitation for adults with multiple sclerosis. Postgrad Med J.

[46] Lacasse Y (2006). Pulmonary rehabilitation for chronic obstructive pulmonary disease. Cochrane Database Syst Rev.

[47] Khan F, Amatya B (2013). Factors associated with long-term functional outcomes, psychological sequelae and quality of life in persons after primary brain tumour. J Neuro-Oncol.

[48] Haussmann A (2020). The influence of Cancer patient characteristics on the recommendation of physical activity by healthcare professionals. Int J Behav Med.

[49] McCourt EM (2021). Evaluation of disaster preparedness and preparedness behaviors among pharmacists: a cross-sectional study in Australia. Prehosp Disaster Med.

[50] Davies C (2020). Interventions for breast Cancer-related lymphedema: clinical practice guideline from the academy of oncologic physical therapy of APTA. Phys Ther.

