# Supplementary material for: Cancer survivorship: understanding the patients’ journey and perspectives on post-treatment needs
Source: BMC Sports Sci Med Rehabil. 2024 Apr 12;16:82. doi: 10.1186/s13102-024-00864-y (PMC11010277; doi:10.1186/s13102-024-00864-y)
Supplement: Supplementary file 1 — Supplementary Material 1. [file 13102_2024_864_MOESM1_ESM.docx]

**Supplementary Information showing illustrative quotes from all thematic areas.**

**Theme: Cast adrift with no direction. Illustrative quotations**

| **E*verything revolves around treatment*** | *Everything revolves around your treatment. But also all of the people that have supported you like your friends, your family and everybody. They’re all there, all the time, while you’re going through your treatment. And the minute you’re finished your treatment it’s not that they completely disappear. But they, everybody wants to go back to being normal. So and they want you to go back to your normal life as well. It’s all good, if you know what I mean. But then there’s nothing. So you’re kind of left you know there’s nothing.*[P2: FG3]  […] *once you’ve finished your cancer journey you seem to be left out on a limb so I find yeah I really struggled* […] *I think yeah there’s definitely a lack of information you know everywhere once you finish your cancer journey.* [P7: FG4]  *[…] once your last treatment was over it was good luck and thanks and don’t let the door hit you on the arse going out the door. Because nobody seemed to care. And it’s not even that I wanted someone to care, I simply wanted information*. [P8: FG6]  *I didn’t have any real support leaving and like the others you just, you’re discharged and its very, its great you’re finished and you’ve got through it but it’s the after bit then and how you manage going forward. You’re left kind of to your own devices, you feel quite isolated and alone. […] You’re dealt with by the professionals to get, you know to get rid of the cancer, give us the treatment, they’re excellent at what they do […] But it’s just you’re really dependent on them at that time […] when you leave them and you’re going back out into the world as such. That piece is missing*. [P4: FG4]  *“ I was chucked into a boat without any oars and shoved out into the ocean*” (P9; FG6).  *You’re left kind of to your own devices, you feel quite isolated and alone. […] You’re dealt with by the professionals to get, you know to get rid of the cancer, give us the treatment, they’re excellent at what they do […] But it’s just you’re really dependent on them at that time […] when you leave them and you’re going back out into the world as such*. [P6: FG4] |
| --- | --- |
| **Panic and fear** | *I’m just praying the days away to be honest, to get to next week. To feel a little bit better. I did a walk today, I don’t know if I said it already but it’s like walking through soup […] I feel very isolated actually to be honest, to be able to talk about this and say my challenge is this, is actually, it’s quite nice, you know what I mean to be able to, that I’m not alone […] You know you at a certain level of, before you start all of this but each step the way its getting worse, it’s going down and down, you’re losing strength, losing stamina, fitness, cardio, ability and you just at some point, you just feel so, I feel old, I feel weak* [P4: FG5]  *I think the biggest fear, like for me the biggest fear is the fear of recurrence and the fear of any little twinge, ache or pain is that something coming back […] my back was sore there a week or a fortnight ago and I went oh my god is it something coming back, is it the exercise, what is it and your mind you know goes into overdrive* [P3: FG4]  *I ended up in hospital a couple of weeks ago in agonising pain and straight away I had cancer back, it was in my bones, it was somewhere else, because your head goes there. […] Because straight away any kind of physical pain and you think it’s back, it’s here […] you become kind of hyper conscious and hyper responsive to every single feeling in your body. And I think that does, it’s very distracting, it’s all consuming at times* [P5: FG6]  *it takes a long time to get past the fear and anxiety, you want guarantees on everything and nobody can give you guarantees and it’s, like you’re basically facing death in the face and going ok so how am I going to do this…that fear will never go away. And it does raise its ugly head every once in a while, I go through peaks and troughs a lot*  [P3: FG1]  *I’m 6 years on now but I still obviously struggle with fear of reoccurrence* […] *I suffered really bad with depression when everything hit me afterwards […]* [P3: FG4]  *I definitely have struggled with that, yeah the loneliness and trying to just, you know you sort of feel like you’re sort of on your own kind of stuff. […] I thought oh my god definitely going through the treatment would be the worst part but I think I’ve nearly found out that the post treatment and I suppose just with everything because it coincided then with covid that it was just, for me I found it really difficult, you know what I mean, I was struggling*. [P3: FG5]  *People don’t understand, my husband hasn’t a clue. So he thinks that once I’m okay and I can get on with the day-to-day stuff then I’m okay. But like you know when you are struggling daily and whatever people just don’t get it […] you just get really withered with life […] when you are feeling so unwell and you can’t do things and you are pulled down, it's very, very hard [..]* [P2: FG7] |
| ***What exercise should I be doing*** | *I certainly felt that there was just no support in any shape or form. And I’m not talking like emotional support or mental support. I’m just talking the physical, what should I be doing* […] *I do think that I should have been able to do a lot more and I should have been doing a lot more but because there was nobody saying to me right ok, you know while you’re going through your therapy do this, you know do 4 walks a week or 3 walks a week or you know do this or do that or do the other. I wasn’t doing anything* [P13: FG8]  […] *when I was having the radiation the radiation oncologist said if you could exercise that would help with the fatigue and I was thinking, he said I know you might think that’s impossible and I was thinking it is impossible. But there was no one out there to help or to know how to go about doing this exercise, how much or I felt I needed guidance […]* [P12: FG6]  *I just feel in my experience there was such a lack of information around exercises, even the nurses had said to me exercise is very important for reducing recurrence etc, I was just still sent off on my own [….] you know exercise is very important but I suppose you know after going through treatment you kind of have no confidence in your body, how much you can do, how much you can push yourself.* [P3: FG4]  *I’m currently on my maintenance treatment […] Little emphasis put on kind of your activity levels and that when you’re discharged and what you’re doing in order to you know improve your general fitness and you know there’s a lot of muscle wasting and that after my transplant. And I found it quite difficult to get information or encouragement, direction even from the hospital. You know you’re basically asked when you go in, you know how your energy levels, you know are you fatigued. And it’s kind of, you know tick the box and that’s it and there’s no follow up.* [P11: FG4]  […] *when I finished [chemotherapy] and I asked, I actually asked where do I go for support and help about exercise, diet to shift the weight I’d gained through chemo etc, etc, etc. And the nurse turned around, now it wasn’t, like it wasn’t her fault, she’s just a product of the system. She said oh that’s got nothing to do with us. She said we only do the chemo and we only deal with the chemo you know* […] [P9: FG6]  *I suppose it was walking that I initially started with. Because that was all I was really kind of capable of and walking was always my thing. And I live next to the sea. But I suppose it maybe would’ve been helpful if there had been you know some kind of guidance as to what you could or couldn’t do. Or what things were more beneficial than others […] in terms of cardiac exercises, things to just get the heart pumping a bit. And there was very little of that.* [P1; FG3]  *I asked my oncologist, you know finishing up [treatment] what’s the dos and don’ts and exercise was, she emphasised very strongly that it was something that you should do, yeah. But like that she quoted the WHO guidelines of, isn’t it 30, what is it 150 minutes a week* […] *but it literally was tiddle off now and do that yourself, do you know what I mean, I think it was. And as I said I had a lot of difficulty doing it, with it. I would have absolutely loved some kind of support like that, absolutely loved it.* [P10: FG1]  […] *inside [hospital] they told me exercise, exercise, so my husband was alive at the time and I had a personal trainer and I was paying this fella forty-five euro an hour I was going about three times a week. And sure I was going to him and I was going to the physiotherapist […] I was paying the same amount of money trying to rectify the damage I was doing with these guys. So then I tried the gym and then I injured myself in the gym. Then I said no, forget about it. So yeah I stopped to be honest with you because I was doing more harm than good.* [P1: FG7] |

**Theme: Everybody is different. Illustrative quotations**

| **Fatigue and brain fog** | […] *your wellbeing is impacted because you’re not in the mood and not in the form to be exercising, moving, you know you’re just exhausted you know and you’re tired and you’re fatigued and you’re wanting to feel the way you did pre all of this but you’re not feeling it.* [P6: FG4]  […] *even from the beginning of diagnoses to going through treatment to getting into a post treatment stage, the fatigue is just horrendous […] I used to be able to go for longer, obviously longer distances and now it’s like your stamina is really affected. Obviously then its fatigue and it kind of gets you down that you can’t get that much done as you used to do. Like even housework, I’m wrecked after a little bit of housework now these days, if I actually get to do it.* [P4: FG5]  *I’m absolutely floored today I’m actually struggling really bad with fatigue today and just like heavy eyes and dull headache. Just totally, my body just feels like heavy and weak and like there isn’t a hope I'd be able to exercise today. I know with fatigue if you exercise it will improve but there’s just some days where your body won’t let you and today is just one of them for me.* [P14: FG7]  […] *the fatigue is still there, the joint pain is still there, whether it’s, you know its side effects from the medication that I’m on, you know it’s just that like your get up and go has got up and gone and hasn’t come back, you know. And I suppose you kind of, I know certainly me mentally I got into a place where it was like I know I need to do this* [exercise] *in order to feel better but in order to feel better I don’t feel I’m capable of doing this. And it was a vicious circl*e […]*.* [P13: FG8]  […] *even from the beginning of diagnoses to going through treatment to getting into a post treatment stage, the fatigue is just horrendous […] I used to be able to go for longer, obviously longer distances and now it’s like your stamina is really affected. Obviously then its fatigue and it kind of gets you down that you can’t get that much done as you used to do. Like even housework, I’m wrecked after a little bit of housework now these days, if I actually get to do it.* [P1: FG5]  *[…] cognitive, fatigue, I suppose it would go into fatigue but certainly my cognitive function, I think has been badly affected, certainly going back into a school setting, multi-tasking was extremely difficult. White noise, massive issue for me* [P4: FG2]  *I’m 36 and fatigue was never a part of life for me before, like I was energiser bunny, constantly on the go. Now I feel like my body is kind of shouting at me to slow down a little bit or it’s stopping me really* […] *I think the fatigue thing is probably what gets me the most. And I suppose I’m kind of saying to myself you know for the first while give yourself a break, you have just had the most difficult year and a half of your life. But then I’m also in the same note beating myself up that why am I not the person I was prior to my diagnoses. And when am I going to get back there. So I’m still convinced I will get back there but then you know I don’t know, like my body is just, it is pretty exhausted at the minute* […] [P2: FG8]  *My ability to put thoughts together, my mind is really slow, extremely. And on the Tamoxifen was really, I couldn’t put a thought process together* [P2: FG5]  *I mean I got a lot of information but coming out of chemotherapy and then a course of radiotherapy I really didn’t, like I let herself my wife read all that stuff and do all the research online you know because I was sleeping so much as well. I just really didn’t have the energy, so that’s where I was at.* [P4: FG7] |
| --- | --- |
| **Nerve pain and neuropathy** | *I remember the first time I experienced nerve pain, it scared me. Like it was very, very scary, it was very painful.* [P3: FG1]  *So first of all I was in nerve pain, like severe nerve pain from when I came out of theatre and I ended up having nerve pain for 6 months afterwards. I had severe disability in my shoulder, like I couldn’t move, I was having nerve spasms, you know muscle tightness* [P5: FG1]  *[…] there was all this weird nerve pain kind of going around into my shoulder and I got a bit of cording under my arm* [P1: FG1]  *the main one is really my right arm, because everything was on the right side. So I can’t really lift it, even many times during the night I have to wake up because my whole arm is numb. And I feel like pins and needles. So I have to tap it, so you know it can come back. And even to hold something, or even use the laptop. My right hand, after a while I have these cramps, where my fingers goes like that. I can’t really move it. […]*[…] *I can still do stuff, but I can’t really hold anything here. I mean you know most of the stuff you need both of your arms and your hands, those are the things I can’t really do anymore.*[P5: FG3]  *I suppose the biggest side effect that I’m struggling with and my treatment doesn’t end now till November but it’s the neuropathy. I would have suffered, I’m on medication for the neuropathy but that’s the biggest kind of thing I’m having to live with. So I would have lost, I work as a fine artist, a photorealist. So fine motor skills are imperative. And I would have lost that 10% of strength you know to be able to actually, you know for example during the entire chemotherapy I couldn’t provide a signature or hold a pencil. I lost the pincher ability. […] I’d be quite cross about it because I feel there was presentations of it from the first round of chemo. So I kind of can’t help but feel that I had an unnecessary high level of it. I very much had it in my face and my hands and my feet. And just walking on carpet is akin to walking on broken glass. And that hasn’t quite changed even though an awful lot has returned to my fingers thank goodness*. [P2: FG6]  *I have peripheral neuropathy, I mean I could keep on going with the side effects that I’ve had from it. My feet have completely changed position, they’ve changed in appearance. So it is extremely difficult to walk. And it's all related back to the chemotherapy. Which is better the cure or the drug, you know, it's hard to tell*. [P1: FG7] |
| **Menopausal symptoms** | *Not only hot flushes, vaginal dryness, mood changes, loss of libido, all those things which are not really, I think chemotherapy units are geared up to give you chemo and deal with neutropenia, nausea and vomiting. And I think there is definitely a deficit there dealing with menopausal symptoms in younger women. All women but particularly in younger women, it’s more problematic.* [P4: FG1]  *I probably found the original, the kind of hot flushes, the hormonal treatments after chemo, that was probably one of the hardest things […] your sleep is off and you’re dealing with I suppose a lot of other stuff and suddenly you’re feeling like this kind of menopausal person, like 10 years ahead of what you ever anticipating to be like that.* [P2: FG1]  *At the start yeah hot flushes were pretty full on, to the point that I’d be changing my pyjamas, changing the bed, that sort of thing in the middle of the night. I figured out that if I took Tamoxifen at night time it was better than during the day* [P3: FG1]  *[…] from day one when I started the treatment, to today. I’ve put up ten kg that’s a lot. Even with all the exercise, the walking, the rowing, I’m not able to get rid of them. I cook my food from scratch. I make my own juice; I don’t buy juice from the shop anymore. I have a juicer. I do everything from scratch. But yea, still stuck with the ten kg, so I don’t know (Laughs).* [P5, FG3]  *My biggest issue is the weight gain, I’ve gained like over 2 ½ stone. I know I was put into full blown menopause after my treatment but it totally was like unexpected* […] *I was on Tamoxifen for a few years but the joint pain was so bad it was inhibiting me from exercising. So I actually made a decision to give it up because for quality of life and being able to exercise*. [P3: FG4]  *I went into a very radical menopause when I started my chemotherapy. And I found that very isolating because no one of my age at the time was going through that and there was no support for that or there wasn’t even any information, nobody told me that this perhaps could happen to me.* [P4: FG4]  *I feel there is a lot of long-term effects, you know you’re very lucky to be here and I’m grateful for it all but a lot of the time you do go back and maybe say to your oncologist and he’s not interested because he’s like you’re here, I’ve made you better. And you know I’m grateful for that. But you still live with a lot of we’ll say pain and you know bone pain, aches, things like that that really impact our lives you know*. [P4: FG4]  *[…] from the treatment I’ve menopause so I’ve the latent affects, I’ve terrible hot sweats and I’m just completely soaked. So like it's just…one thing into the other like. And then I have been diagnosed with Addison’s so like that I’m on steroids so I’m watching what I’m eating but I’m just blown up like you can see my face and my neck and every bit of me is swollen, I’m puffy, I can’t wear my rings. I’m very, very uncomfortable. You don’t feel like yourself and when you have that extra I don’t know I feel like a Michelin man blown up with all the steroids and it's very difficult to move.* [P2: FG7]  *[…] Hot drinks as soon as I’ve had a hot drink I’ll start sweating. And yeah kicking off the big clothes and then putting them back on, makes for a restless night’s sleep. And basically I’m walking around in summer attire almost all the time and it's freezing outside. I’m always wearing shorts the whole time. So that’s about the only thing.*[P4: FG7]  *I had phenomenally aching joints, as in all over my body. And the physio said it is like arthritis. And I still have it, particularly in my feet and hands. We don’t know if its from the treatment or if it’s from, you know I’d be going through severe menopause now, we don’t know what it is. So again that was one I really struggled with […] I get a lot of hot sweats now and they really keep me awake at night actually […] yeah the nights, I wake up a lot at night with hot sweats and things like that.* [P1: FG9] |
| ***Personalised exercise program*** | *[…] you can’t expect the same exercise program to be given just because we both have a cancer diagnoses in common. It’s not doing anybody any favours by both of us doing the same program. So I think that’s why it’s really important that its tailored and its progress orientated as opposed to goal orientated. And I think that is where that’s the key to it* [P2: FG1]    *everybody is different, their levels of fitness are different. And as you say if they’ve had different surgeries and that, how debilitated they are after the surgery […] I think we have to give patients, you know instead of just saying exercise is important, we have to kind of get them assessed and see what exercise is appropriate for them*. [P4: FG1]  […] *for people who are, you know who are very elderly. Do a really easy one. For people who are you know, or for somebody who’s you know, who’s really knackered and in a lot of pain. Do a really easy one. But for somebody who’s kind of feeling okay, but just tired. You know where you can adjust it to how the person is themselves.* [P1: FG3]  […] *if you had to do it then, then with somebody watching you for a period of time. Just to get you going, do you know what I mean; I think that would be hugely beneficial.* (P2: FG3]  *I think something like a program, you know because in my experience the nurses said to me, you know exercise is very important but I suppose you know after going through treatment you kind of have no confidence in your body, how much you can do, how much you can push yourself. So actually a step by step program, you know with basic exercises I just feel would be really helpful. And actually breaking it down into like what you’re doing around the aerobic, the strength and flexibility*. [P2: FG4]  *I find that this program is excellent and if this was available when you were coming out of your treatment it would be brilliant for people, just building confidence, targeting you and targeting what maybe different surgery because I know everyone has different cancers.* [P4: FG4]  […] *if somebody was able to assess where I am at the moment with regards to the limitations and actually design something for me that’s not going to damage my or you know worsen any of my joints or mobility or something like that but actually improve it. It gives you a goal every day, it gives you somebody who knows what they’re doing so you can actually. And if you can’t achieve it that somebody can give you some feedback as in this could change, you could do it this way. And I think that’s just, I think that would really help as in kind of even mentally that you’re getting better and you can feel it. If you can feel something improving on a weekly basis then you know it really gives you a mental lift. You know what I mean it kind of propels you forward*. [P1: FG5]  *I’m just really grateful that there’s something like this that we can go forward with*. [P4: FG7]  *I’m enjoying the fact that you know have a piece of paper but you know I’ve my own notes written and so that I can actually do this. *** [Exercise physiologist] has made sure that I’m actually doing them right. So to me that’s a huge thing. I know what I’m doing and I’m hopefully, I can bring them on holidays with me. So that I won’t be behind the schedule you know.* [P1: FG7]  […] *it’s not like going into a gym and some young fella putting a plan together for you and you saying no I can’t do that and he’s like can’t or won’t, can’t or won’t. Whereas with *** [physical exercise trainer] he very much understands where you’re coming from. And* *he would be the one who would say to you start slow, we build up, don’t be going hell for leather thinking that you’re doing the right thing, because you won’t stick at it. You know so I’m very happy with the program that he has put together for me*. [P1: FG8]  […] *when I initially met **** [exercise physiologist] *he gave me the opportunity to go through my previous exercise history and gauge where I’m at with my fitness level. So it wasn’t just like a one for all program, which was brilliant. So yeah he took everything into consideration and came up with a fantastic plan*. [P2: FG8] |

**Feedback on the personalised exercise programme**

| ***Personalised exercise program*** | *[…] you can’t expect the same exercise program to be given just because we both have a cancer diagnoses in common. It’s not doing anybody any favours by both of us doing the same program. So I think that’s why it’s really important that its tailored and its progress orientated as opposed to goal orientated. And I think that is where that’s the key to it* [P2: FG1]    *everybody is different, their levels of fitness are different. And as you say if they’ve had different surgeries and that, how debilitated they are after the surgery […] I think we have to give patients, you know instead of just saying exercise is important, we have to kind of get them assessed and see what exercise is appropriate for them*. [P4: FG1]  […] *for people who are, you know who are very elderly. Do a really easy one. For people who are you know, or for somebody who’s you know, who’s really knackered and in a lot of pain. Do a really easy one. But for somebody who’s kind of feeling okay, but just tired. You know where you can adjust it to how the person is themselves.* [P1: FG3]  […] *if you had to do it then, then with somebody watching you for a period of time. Just to get you going, do you know what I mean; I think that would be hugely beneficial.* (P2: FG3]  *I think something like a program, you know because in my experience the nurses said to me, you know exercise is very important but I suppose you know after going through treatment you kind of have no confidence in your body, how much you can do, how much you can push yourself. So actually a step by step program, you know with basic exercises I just feel would be really helpful. And actually breaking it down into like what you’re doing around the aerobic, the strength and flexibility*. [P2: FG4]  *I find that this program is excellent and if this was available when you were coming out of your treatment it would be brilliant for people, just building confidence, targeting you and targeting what maybe different surgery because I know everyone has different cancers.* [P4: FG4]  […] *if somebody was able to assess where I am at the moment with regards to the limitations and actually design something for me that’s not going to damage my or you know worsen any of my joints or mobility or something like that but actually improve it. It gives you a goal every day, it gives you somebody who knows what they’re doing so you can actually. And if you can’t achieve it that somebody can give you some feedback as in this could change, you could do it this way. And I think that’s just, I think that would really help as in kind of even mentally that you’re getting better and you can feel it. If you can feel something improving on a weekly basis then you know it really gives you a mental lift. You know what I mean it kind of propels you forward*. [P1: FG5]  *I’m just really grateful that there’s something like this that we can go forward with*. [P4: FG7]  *I’m enjoying the fact that you know have a piece of paper but you know I’ve my own notes written and so that I can actually do this. *** [Exercise physiologist] has made sure that I’m actually doing them right. So to me that’s a huge thing. I know what I’m doing and I’m hopefully, I can bring them on holidays with me. So that I won’t be behind the schedule you know.* [P1: FG7]  […] *it’s not like going into a gym and some young fella putting a plan together for you and you saying no I can’t do that and he’s like can’t or won’t, can’t or won’t. Whereas with *** [physical exercise trainer] he very much understands where you’re coming from. And* *he would be the one who would say to you start slow, we build up, don’t be going hell for leather thinking that you’re doing the right thing, because you won’t stick at it. You know so I’m very happy with the program that he has put together for me*. [P1: FG8]  […] *when I initially met **** [exercise physiologist] *he gave me the opportunity to go through my previous exercise history and gauge where I’m at with my fitness level. So it wasn’t just like a one for all program, which was brilliant. So yeah he took everything into consideration and came up with a fantastic plan*. [P2: FG8] |
| --- | --- |
