# Supplementary material for: Cancer survivorship: understanding the patients’ journey and perspectives on post-treatment needs
Source: BMC Sports Sci Med Rehabil. 2024 Apr 12;16:82. doi: 10.1186/s13102-024-00864-y (PMC11010277; doi:10.1186/s13102-024-00864-y)
Supplement: Supplementary file 2 — Supplementary Material 2. [file 13102_2024_864_MOESM2_ESM.docx]

**Table. s1 Supplementary Information: Cancer Rehabilitation Focus Group coding and numbers: The table below illustrates the actual number of participants that joined each focus group interview. The codes are listed here as these codes link comments made by each participant during the interview.**

| **Focus Group Number and Date** | **Code** |
| --- | --- |
|  | P1:FG1 |
|  | P2:FG1 |
| **FG1** | P3:FG1 |
| **01/07/2021** | P4:FG1 |
|  | P5:FG1 |
|  | P1:FG2 |
|  | P2:FG2 |
| **FG2** | P3:FG2 |
| **06/07/2021** | P4:FG2 |
|  | P5:FG2 |
|  | P6:FG2 |
|  | P1:FG3 |
| FG3 | P2:FG3 |
| 07/07/2021 | P3:FG3 |
|  | P4:FG3 |
|  | P5:FG3 |
|  | P1:FG4 |
| **FG4** | P2:FG4 |
| **12/07/2021** | P3:FG4 |
|  | P4:FG4 |
|  | P1:FG5 |
| **FG5** | P2:FG5 |
| **17/08/2021** | P3:FG5 |
|  | P1:FG6 |
|  | P2:FG6 |
|  | P3:FG6 |
| **FG6** | P4:FG6 |
| **18/08/2021** | P5:FG6 |
|  | P6:FG6 |
|  | P1:FG7 |
| **FG7** | P2:FG7 |
| **12/10/2021** | P3:FG7 |
|  | P4:FG7 |
| **FG8** | P1:FG8 |
| **19/10/2021** | P2:FG8 |
| **FG9** | P1:FG9 |
| **02/11/2021** | P2:FG9 |
