# Supplementary material for: Cancer survivorship: understanding the patients’ journey and perspectives on post-treatment needs
Source: BMC Sports Sci Med Rehabil. 2024 Apr 12;16:82. doi: 10.1186/s13102-024-00864-y (PMC11010277; doi:10.1186/s13102-024-00864-y)
Supplement: Supplementary file 3 — Supplementary Material 3. [file 13102_2024_864_MOESM3_ESM.docx]

**Appendix 1: Focus group topics guide for participants.**

**The purpose of the focus group is to understand your opinion as a “Cancer Survivor” about the changes to health and fitness that you may have experienced during and after completing your treatment and what resources and support you believe will be helpful towards supporting those needs.**

**Specifically we would like you to share your opinion on**

- **What programme and resources are needed to improve your health, fitness and wellbeing**

**Towards this goal we will focus our discussion under three broad topics:**

1. Information and Resource needs
2. Physical, health related fitness, and Psycho-social needs
3. Exercise programme and support needs

Guide questions or prompts:

**Topic 1: 20 minutes**

**Information and Resources needs –**

What information and resources do you need to be able to manage your health after treatment? How would you like/prefer to have access to information

**Topic 2: 45 minutes**

1. **Post treatment Physical and health related needs – 45 minutes**
2. Have you experienced any of the following **after completing your treatment**:

**Pain,** **Fatigue, Tiredness, Swelling, Loss of balance, Reduced flexibility, Reduced strength, Reduced mobility,** breathlessness, claudication pain, arrhythmia, high blood pressure, dizziness, weight gain or weight loss, lack of sleep

1. How do you think these side effects of treatment has **affected your health and fitness and physical function?**
2. **Did you experience any changes in your sense of taste or smell?**

**B. Psychological, emotional and social needs**

Could you tell us about any psychological effects that you have experienced since finishing treatment?

Prompts: fear, anxiety…What were the main concerns behind the fear or worry?

1. **Quality of Life/ Well-Being – 10 Minutes**

Could you tell about the how the cancer treatment has affected your overall wellbeing and quality of life? Have you felt calm and peaceful? How often do you relax?

**Topic 3: 15 minutes**

**Developing a Personalised Exercise Rehabilitation Programme –**

Do you want to participate **in a long term personalised rehabilitation programme** that will help you improve your physical fitness and help to address these symptoms?

Do you think this will be beneficial?
